# Development of Managerial Key Performance Indicators for A Hospital Pharmacy Digital Dashboard

**DOI:** 10.22037/ijpr.2019.1100822

**Published:** 2019

**Authors:** Arezoo Dehghani Mahmodabadi, Mostafa Langarizadeh, Mohammad Hossein Mosaddegh Mehrjardi, Sima Emadi

**Affiliations:** a *Department of Health Information Technology and Management, School of Public Health, Shahid Sadoughi University of Medical Sciences, Yazd, Iran. *; b *Health Management and Economics Research Center, School of Health Management and Information Sciences, Iran University of Medical Sciences, Tehran, Iran. *; c *Department of Toxicology, Faculty of Pharmacy, Shahid Sadoughi University of Medical Sciences, Yazd, Iran. *; d *Department of Computer Engineering, Faculty of Enginearing, Islamic Azad University, Yazd Branch, Yazd, Iran.*

**Keywords:** Key performance indicator, Hospital pharmacy, Dashboard, Pharmacy management

## Abstract

Pharmaceutical performance is a critical factor in the hospital operation. Hospital pharmacy activities require retrieving, processing, comparing, and updating the information. Dashboards are new tools that can track key performance indicators by displaying information to managers in order to improve the performance of the hospital pharmacy. We conducted this study to determine the performance indicators of hospital pharmacies in Iran. This qualitative research was conducted in 2016. The participants were hospital pharmacists and hospital managers. A semi-structured questionnaire was constructed to determine key performance indicators of the hospital pharmacy department. The questionnaire was used in face-to-face interviews and focus groups. The data were analyzed using Framework analysis. The indicators comprised three domains, including managerial indicators (satisfaction, education, staffing, and department management), clinical indicators (patient safety), and financial indicators (income, costs, and financial utilization). Traditionally, pharmacy services included provision and distribution of drugs in the hospital; however, today, with an increase in the complexity and diversity of the drugs, hospital pharmacy services include diverse fields beyond clinical affairs. It could be concluded that pharmaceutical performance has a vital role in successful hospital management. Hospital pharmacy management is not possible without monitoring performance indicators.

## Introduction

Healthcare is a repository of clinical, operational, and financial data. The data are stored in disparate systems without any coordination among data owners. However, with discrete systems, considerable time and energy are spent on the creation, distribution, and analysis of the reports (1). For this reason, there is an increasing demand for new information systems which can organize the reporting process (2).

Hospital pharmacy is one of the most important hospital departments responsible for controlling the drug distribution and consumption process. It warrants timely access to drugs, drug consumption safety, and effective and efficient drug use. Hospital pharmacy activities require information retrieving, processing, comparing, and updating (3). In the pharmacy, the data are provided from different sources, such as the pharmacy information system, financial information system, hospital information system, and personnel information system. On the other hand, with a greater focus on medical treatment, cost reduction, and quality improvement, pharmacy managers need to monitor the department data precisely. They can improve their department activities through using accurate and timely data to make better decisions (4, 5). 

Automatic monitoring of hospital pharmaceutical services results in the reduction of drug adverse effects, improvement of the cost effectiveness of medication management, and better management of the hospital pharmacy. Health care organizations are increasingly pressed for better performance while dealing with decreased resources and increased expenditure more than ever, indicating that the health industry requires new approaches for resource management to optimize its current situation (6, 7).

In order to improve the hospital pharmacy performance, patient safety, cost effectiveness, and economic efficiency, pharmacy managers need to use new tools for effective and efficient data collection. With this information, key challenges of quality improvement programs (information collection from various sources, integration, and delivery to managers) would be addressed. Business intelligence introduces a new technology to overcome such challenges that can track key performance indicators and improve the department performance through delivering this information to managers (4, 8-11).

Key Performance Indicators (KPIs) are the criteria defined for performance objectives so that an organization can monitor its progress towards predefined targets (12). A set of measurable quantitative metrics is used to measure and compare the performance in terms of achieving the organizational strategic and operational goals. It is very important to select KPIs based on organizational goals (12, 13).

Recently, healthcare organizations are persuaded to look for a set of manageable performance indicators to use for timely decision making beyond traditional domains of inefficient financial data, limited clinical indicators, and confusing satisfaction criteria (14). These indicators not only include financial measurements but also comprise inter-organizational process indicators (such as the clinical outcome), and developmental and educational indicators (*e.g.* staff achievement and information technology). The managers of healthcare organizations have experienced a growth in performance indicators with a new focus on key operational issues, such as patient safety and care management that finally affect the financial performance (15-17).

Dashboards are a visual display of key performance indicators that offer an at-a-glance window into the overall business performance and can display integrated data from various sources in a unified and interactive manner (12, 13). Drill-down into different levels of information, ability to identify trends, real-time performance monitoring, and customization for different users are some capabilities of dashboards (1, 18). The first step in dashboard development is selecting key performance indicators and determining the underlying relationship between them (19). However, determining key performance indicators is the key to dashboard success (20, 21). With an increasing demand for improved hospital pharmacy performance, the importance of a set of indicators to assess the performance of this department is evident, considering the differences between the current and ideal situations in the pharmacy information system, including documentation of pharmaceutical information, access to patient information and diagnosis, evaluation of the staff workload, etc. (3, 22 and 23).

In this paper, we tried to determine suitable performance indicators for Iranian hospital pharmacies. 

## Experimental

 This qualitative research with a content analysis approach was conducted in 2016. The participants included hospital pharmacy managers and hospital managers of state (4 hospitals), private (2 hospitals) and charity (1 hospital) hospitals in Yazd. Data were collected from all populations. Based on a review of the literature, a semi-structured questionnaire was constructed to determine key performance indicators of the hospital pharmacy department. The questionnaire was used in face-to-face interviews and focus group discussion. The focus group discussion was held in a big hospital with the participation of the hospital managers and hospital pharmacy managers. The interviews started with general questions followed by detailed questions after the participants’ responses. The interviews were recorded after obtaining the participants’ consent. Each interview took at least 30 to 50 min, and data saturation was achieved after 15 interviews.

The interviews were transcribed and analyzed thematically. The responses were coded and collected to create themes. A list of coded concepts was developed to characterize key performance indicators for pharmacies. The codes were then grouped to delineate emerging themes and their relationships. Analyses were done to identify heading and subheading relationships. Finally, the results were returned to the participants for validation and accreditation. We masked the identifying information in the excerpts of interview transcripts to protect the anonymity of the participants. 

## Results

In this study, hospital pharmacy key performance indicators were determined. The participants included 11 hospital pharmacy managers and 4 hospital managers. Performance indicators were defined in different levels for dashboard display to follow different objectives. The main KPIs or first-layer indicators were general and measured the overall performance of the pharmacy. In the next layers, there were more detailed indicators in comparison with the first layer. KPIs in the first and second layers are shown in Table 1.

The indicators comprised three domains of managerial indicators (including satisfaction, education, staffing, and department management), clinical indicators (including patient safety) and financial indicators (including income, cost and financial utilization). Some participants’ responses are presented in the following.

*Managerial performance indicators*

About satisfaction indicators, a hospital pharmacy manager (pm1) said, ″The hospital manager’s satisfaction with the pharmacy performance is very important and is often achieved with cost reduction.″ ″The physician or the department sends satisfaction or dissatisfaction reports directly to the pharmacy or indirectly to the hospital manager,″ said another participant (pm5). 

As for education, a hospital pharmacy manager (pm3) said, ″Education is better to be different according to the job level in the hospital pharmacy and to be specific to the hospital pharmacy management, not general pharmacology courses.″

Regarding pharmacy management, a hospital manager (hm4) said, ″Assessment of unusable and expired drugs and equipment is very important, even in other departments or operation rooms, but unfortunately there is no accurate statistics about them. It is a form of hidden costs because even if the costs are compensated by the insurance, they are subject to deductions. 

A hospital pharmacy (pm10) said, Since drugs are sent to wards on a daily or weekly basis, time is not very important for sending routine drugs but is crucial for emergency drugs. 

Although a hospital information system provides some indicators on the consumption rate, the participants discussed the need for various reports in this regard. Assessment of monthly and weekly drug consumption even with more details based on the disease and diagnosis is necessary for managers in different wards, said one of the participants (hm2).

*Clinical performance indicators*

The participants also stated that hospital information systems in their hospitals had various capabilities. One of the participants (pm8) said, As there is no documentation in the hospital information system about drug allergies or drug interactions, it is not possible to assess these items but there are very important clinical indicators. 

*Financial performance indicators*

Most participants agreed that although there were drug bills in the hospital information system, there were no special reports. All drug bills are available in the pharmacy information system but it is very useful to have reports based on the ward on a monthly basis, said one of the participants (hm1). Another participant (pm11) stated, It is very important to control and assess drugs and equipment bills in intensive care units, operation rooms, and emergency wards. 

About the sales rate, a hospital manager (pm7) said, In addition to common drugs, discounts, gifts, and promotions from drug selling companies should also be calculated. 

Most participants agreed drugs should be purchased based on their expiry date. A hospital manager (hm2) said, “Drug purchase must be based on the consumption rate and expiry date to decrease the costs of warehouse handling.” 

About insurance deductions, a pharmacy manager (pm6) said, Deductions of each ward must be clear to control the overall deduction. 

A pharmacy manager stated that the profit of selling drugs and equipment must be clear. “The profit of selling drugs and equipment must be clear. Even the profit of selling domestic drugs must be clear. This information can be used to save the costs,” said pm3.

**Table 1:** First and second layer KPIs

**Performance domains**	**General indicators**	**Detailed indicators**
Managerial performance indicators	Satisfaction indicators	Other wards’ satisfaction
		Physician satisfaction
		Management satisfaction
	Education indicators	Access to the Internet
		Access to E-guideline
		Access to hospital pharmaceutical guidelines
		Access to drug software
	Staffing indicators	Number of pharmacists
		Number of pharmacy technicians
	Department management indicators	Number of expired and out-of-date drugs
		Number of expired and out-of-date devices
		Number of prescriptions not completely filled
		Time to sending emergency drug orders
		Consumption rate of drugs and equipment in various periods
		Consumption rate of drugs and equipment according to diagnosis
Clinical performance indicators	Patient safety indicators	Rate of drug availability
		Control of drug allergies rate
		Control of drug contraindications
		Rate of expired drugs returned from wards
Financial performance indicators	Income indicators	Total drug billing according to various periods
		Total drug billing according to wards
		Total drug billing according to intensive care unit, emergency ward and operation room
		Total drug billing according to type of drug and equipment
		Reimbursement rate
		Total sale of special drugs
		Total sale rate according to drugs(most consumed drugs)
		Calculation of drug storage rate based on drugs and various periods
		Rate of discount and gifts from drug companies
	cost indicators	Costs of drugs based on consumption rate and storage time
		Most expensive drugs in various periods
		Total purchase rate of equipment
		Total deductions based on wards
		Total costs of expired drugs
		Total personnel salary
		Total personnel bonus
	Financial Utilization indicators	Sales profit based on drugs, equipment, being imported or domestically produced, hoteling
		Total income of drugs and equipment
		Staffing ratio on total income
		Income returned from out-of-pocket payments
		Income returned from reimbursement

## Discussion

Based on the results, the main performance domains in the hospital pharmacy were managerial, clinical, and financial domains. Pharmacy services traditionally included provision and distribution of drugs in the hospital but today, with an increase in the complexity and diversity of the medicines, hospital pharmacy services include diverse fields beyond clinical affairs (24, 25).

Physician, ward, and management satisfaction were emphasized as satisfaction indicators; however, patient satisfaction was regarded as a subset of physician satisfaction due to lack of knowledge.

Access to the Internet, E-literature, electronic guidelines of hospital drugs, and pharmaceutical software were emphasized as educational indicators; similarly, other studies have also shown the importance of these factors in clinical pharmacy (26). In the pharmacy management domain, because of increased pharmaceutical expenses and the necessity of spending the resources in the best possible way, management of expired drugs, incomplete prescriptions, time to sending emergency drugs, drug return rate, and drug and equipment consumption rate in various periods based on the diagnosis were regarded as managerial indicators. Imani mentioned these indicators in evaluating the financial and economic performance of the pharmacy (27, 28). These indicators, in addition to a negative impact on the pharmacy financial outcome, could have deleterious effects on patient safety. Pharmacy management depends on the management of these indicators to manage costs and clinical care procedures, and monitor quality services. Different studies have considered financial indicators as the main component of hospital indicators, and have mentioned total net profit, staffing cost, total drug and equipment cost, sales profit for drugs and equipment, assurance reimbursement, and expired drugs costs as financial indicators of the pharmacy department (12, 27-29).

Due to the failure of information systems and lack of information about some indicators, a number of indicators were not included in the dashboard development. 

## Conclusion

Pharmaceutical performance is a critical factor in the hospital performance. Pharmaceutical services are central to patient care and play an important role in the hospital expenditure (27). If the system is not effective, the quality of patient care is affected, resulting in increased hospital costs. The results of several studies demonsrate that hospital management is not possible without monitoring performance indicators (30). Dashboards are a tool for continuous monitoring of the performance of healthcare organizations, and indicators are essential to the success of dashboards. They are linkage points between the organization and the technical architecture (17, 18 and 31). Based on these indicators, management dashboards can be designed for hospital pharmacies. However, dashboard development has multiple steps and determination of the indicators is one of them. 
